# Analysis of DNA methylation patterns in the tumor immune microenvironment of metastatic melanoma

**DOI:** 10.1002/1878-0261.12663

**Published:** 2020-03-21

**Authors:** Shamik Mitra, Martin Lauss, Rita Cabrita, Jiyeon Choi, Tongwu Zhang, Karolin Isaksson, Håkan Olsson, Christian Ingvar, Ana Carneiro, Johan Staaf, Markus Ringnér, Kari Nielsen, Kevin M. Brown, Göran Jönsson

**Affiliations:** ^1^ Division of Oncology and Pathology Department of Clinical Sciences Faculty of Medicine Lund University Lund Sweden; ^2^ Division of Cancer Epidemiology and Genetics National Cancer Institute Washington DC USA; ^3^ Department of Surgery Skåne University Hospital Lund Sweden; ^4^ Department of Oncology Skåne University Hospital Lund Sweden; ^5^ Department of Biology National Bioinformatics Infrastructure Sweden Science for Life Laboratory Lund University Lund Sweden; ^6^ Department of Dermatology Helsingborg General Hospital Sweden

**Keywords:** DNA methylation, immune cells, melanoma, pan‐cancer, tumor microenvironment

## Abstract

The presence of immune cells in the tumor microenvironment has been associated with response to immunotherapies across several cancer types, including melanoma. Despite its therapeutic relevance, characterization of the melanoma immune microenvironments remains insufficiently explored. To distinguish the immune microenvironment in a cohort of 180 metastatic melanoma clinical specimens, we developed a method using promoter CpG methylation of immune cell type‐specific genes extracted from genome‐wide methylation arrays. Unsupervised clustering identified three immune methylation clusters with varying levels of immune CpG methylation that are related to patient survival. Matching protein and gene expression data further corroborated the identified epigenetic characterization. Exploration of the possible immune exclusion mechanisms at play revealed likely dependency on *MITF* protein level and *PTEN* loss‐of‐function events for melanomas unresponsive to immunotherapies (immune‐low). To understand whether melanoma tumors resemble other solid tumors in terms of immune methylation characteristics, we explored 15 different solid tumor cohorts from TCGA. Low‐dimensional projection based on immune cell type‐specific methylation revealed grouping of the solid tumors in line with melanoma immune methylation clusters rather than tumor types. Association of survival outcome with immune cell type‐specific methylation differed across tumor and cell types. However, in melanomas immune cell type‐specific methylation was associated with inferior patient survival. Exploration of the immune methylation patterns in a pan‐cancer context suggested that specific immune microenvironments might occur across the cancer spectrum. Together, our findings underscore the existence of diverse immune microenvironments, which may be informative for future immunotherapeutic applications.

## Graphical abstract

The importance of the tumor immune microenvironment has been highlighted in multiple cancers, but information regarding the contribution of individual immune cell types is lacking for melanoma. In this study, we have sought to fill the gap by identifying distinct DNA methylation patterns for specifc immune cells. These were corroborated by other molecular correlates and found to be shared across other types of solid tomors.

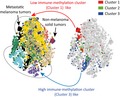

**Abbreviations**
Bp: base pair
CD: cluster of differentiation
CNS: central nervous system
CpG: cytosine phosphate guanine
DHS: DNase hypersensitivity sites
DMFS: distant metastasis‐free survival
DSS: disease‐specific survival
FDR: false discovery rate
GDSC: genomics of drug sensitivity
ICI: immune checkpoint inhibitor
IHC: immunohistochemical
MM: metastatic melanoma
mRNA: messenger RNA
NK cell: natural killer cell
SCNA: somatic copy number alteration
TCGA: The Cancer Genome Atlas
TIL: tumor‐infiltrating lymphocyte
TLS: tertiary lymphoid structures
tSNE: t‐distributed stochastic neighbor embedding
TSS: transcription start site

## 1 Introduction

Metastatic cutaneous melanoma is the most aggressive form of skin malignancy with poor patient survival (Surveillance, 2019). Based on advancement in targeted therapies and immunotherapies, survival outcome has, however, increased considerably (Chapman *et al.*, 2011; McDermott *et al.*, 2014; Robert *et al.*, 2014). Immune checkpoint inhibitors (ICIs), such as CTLA4 and PD1/PDL1 blockade, that reactivate tumor‐associated effector T cells have demonstrated dramatic clinical efficacy and provide long‐term survival in a substantial fraction of patients (Hodi *et al.*, 2010; Weber *et al.*, 2015, 2017; Wolchok *et al.*, 2017). Multiple studies have identified predictive biomarkers for therapy response that include activity of intratumor immunological effector cells (Riaz *et al.*, 2017; Yuan *et al.*, 2011), tumor mutational burden (Riaz *et al.*, 2017; Van Allen *et al.*, 2015), mismatch repair deficiency (Le *et al.*, 2017b; Viale *et al.*, 2017), tumor aneuploidy (Davoli *et al.*, 2017), PDL1 levels (Long *et al.*, 2016; Patel and Kurzrock, 2015), and intestinal microbiota (Gopalakrishnan *et al.*, 2018; Matson *et al.*, 2018). However, the still large fraction of nonresponding patients (Hodi *et al.*, 2010; Wolchok *et al.*, 2017) warrants further understanding of various factors determining primary resistance to ICIs, including the role of the tumor microenvironment.

The tumor microenvironment is a complex mixture of malignant and nonmalignant cells that includes immune cells. Clearly, the microenvironment of melanoma tumors, especially immune cells, plays an important role in determining progression (Passarelli *et al.*, 2017; Tucci *et al.*, 2018; Villanueva and Herlyn, 2008). Single‐cell RNA sequencing of melanoma tumors has confirmed that a complex mixture of immune T‐cell subtypes exists within a tumor (Jerby‐Arnon *et al.*, 2018; Sade‐Feldman *et al.*, 2018). In addition, bulk transcriptomic analyses have been utilized to explore the immune microenvironment from melanoma tumors, demonstrating that tumors with increased expression of genes involved in a diverse range of immune systems have increased survival (Angelova *et al.*, 2015; Bindea *et al.*, 2013). Current methods to decipher the immune landscape in tumors are based on the transcriptome (Angelova *et al.*, 2015; Bindea *et al.*, 2013). However, global DNA methylation patterns are largely associated with cellular lineage and show higher distinction between cellular lineages than mRNA expression in blood and skin lineages (Bock *et al.*, 2012). Moreover, DNA methylation has been demonstrated to resolve cell of origin of peripheral blood cells (Houseman *et al.*, 2012) and cell‐free DNA (Moss *et al.*, 2018), and was introduced as a complementary approach to classify central nervous system (CNS) tumors (Capper *et al.*, 2018). Nevertheless, DNA methylation has not been extensively exploited to determine the immune lineages occurring in the microenvironment of melanoma. Therefore, we created DNA methylation signatures that reflect a wide range of tumor‐associated immune cell subsets. These signatures grouped melanoma tumors into three clusters with distinct clinical and molecular properties that were re‐identified in independent data. Further molecular exploration revealed several immune exclusion mechanisms activated in tumors. Moreover, an immune‐rich microenvironment consisting of a broad range of immune cells was observed across several cancer types. In conclusion, DNA immune methylation signatures harbor significant clinical and biological information.

## 2 Materials and methods

### 2.1 Patient sample information

The study was approved by the Regional Ethics Committee at Lund University (Dnr. 191/2007 and 101/2013). The experiments were undertaken with the understanding and written consent of each subject. The study methodologies conformed to the standards set by the Declaration of Helsinki. The sample cohort consisted of 214 melanoma tumors and was obtained at the Department of Surgery at Skåne University Hospital. This is a historic cohort collected before the era of ICIs and targeted therapies.

### 2.2 Sample preparation and bisulfite conversion

DNA extraction from patient samples was done as described previously (Harbst *et al.*, 2014) and bisulfite‐converted using EZ DNA Methylation Kit (Zymo Research Europe, Freiburg, Germany) according to the manufacturer's instructions and analyzed using Illumina Infinium MethylationEPIC BeadChip array. Methylation data are available in Gene Expression Omnibus with accession number http://www.ncbi.nlm.nih.gov/geo/query/acc.cgi?acc=GSE144487.

### 2.3 Immune cell type‐specific CpG set for metastatic melanoma

We selected two immune cell type‐associated gene signatures (Angelova *et al.*, 2015; Tirosh *et al.*, 2016) to identify immune cell types in melanoma tumors. Next, we combined both gene signatures, and after removing conflicting genes (genes belonging to different cell types in each signature), we obtained a total of 920 genes associated with 30 immune cell subsets. Next, we selected corresponding promoter CpGs (< 1500 bp from TSS) of these genes, since their association with the transcriptional regulation of a gene is well known and thus more likely to be lineage‐specific. After mapping the gene signatures with the list of CpG probes shared between 450K and EPIC platforms, we found 4228 promoter CpGs belonging to 744 genes and associated with 30 immune cell subsets.

The CpGs were then further filtered using the following criteria:

1. *Selecting the most differentially methylated CpGs across reference immune cells:* We dichotomized the β‐values into robust methylation bins, as unmethylated (β < 0.3) and methylated (β ≥ 0.3). We then selected CpGs that have significantly different proportions of methylated and unmethylated signals among the reference immune cells using Fisher's exact test and an FDR < 0.01.
2. *High methylation in nonimmune cells:* To ensure that any methylation difference we observe is likely coming from immune cells and not from other cells present in the microenvironment, we further shortlisted CpGs with a high percentage and level of methylation (β > 0.7 in > 98% of samples) among nonimmune normal cells and melanoma cell lines.
3. *Forming gene–CpG pairs:* We wanted to ensure that any single gene is not over‐represented through the presence of multiple CpGs. We therefore selected the most significant CpG for each gene from Fisher's exact test in step 1.

The selection processes resulted in 67 gene–CpG pairs belonging to 21 immune cell populations.

### 2.4 Immune cell type‐specific CpG set for nonmelanoma TCGA pan‐cancer cohorts

The CpG selection procedure was identical to the process we followed for metastatic melanoma (MM) tumor cohorts except step 2. At step 2, we filtered the immune CpGs against methylation profiles of matching tumor cell lines from Genomics of Drug Sensitivity (GDSC) database and selected CpGs for further analyses if they had shown high level and percentage of methylation in the tumor cell lines (β > 0.7 in > 90% of samples). Here, we had to relax the sample selection criteria since for a number of GDSC tumor types a minority of cell lines were displaying a methylation pattern that deviated from the majority of cell lines of the tumor type.

### 2.5 Immune methylation centroid‐based classification

Immune methylation centroid‐based classification was performed by correlating sample methylation profiles across centroid CpGs to each cluster centroid (Table S3) and then selecting the cluster that reported highest correlation (τ_Kendall_). If no cluster displayed correlation ≥ 0.3, then the sample was annotated unclassified.

### 2.6 Immune cell type‐specific methylation score calculation

Immune cell type‐specific methylation scores were calculated using the matching CpGs from the 67 CpG set for MM cohorts and by taking median methylation value of all CpGs belonging to a specific immune cell type. For non‐MM solid tumor cohorts, the process is almost identical to MM but here cohort‐specific immune CpG sets were used for score calculation.

### 2.7 PTEN promoter hypermethylation calculation

We selected promoter CpGs for the *PTEN* gene that are located at the DNase hypersensitivity sites (DHS) of the promoter, as the *PTEN* promoter contained a complex set of CpGs on the Illumina 450K array. Next, we called *PTEN* hypermethylation in tumors if more than 10% of the DHS promoter CpGs are hypermethylated (β > 0.7) and median β‐value for all *PTEN* promoter CpGs for the corresponding tumor is above 0.5.

### 2.8 Statistical analyses and calculations for immune cell type methylation and gene expression scores

All statistical and bioinformatics analyses were performed in R. For comparing numerical values, we used Spearman and Kendall correlation. Comparisons between two groups were performed using Mann–Whitney *U*‐test/Wilcoxon rank‐sum test, and for more than two groups, we used Kruskal–Wallis test. For survival analyses, we used univariate and multivariate Cox regression.

Both methylation and gene expression scores were calculated by median methylation/expression of CpGs/genes belonging to each immune cell type. We used median‐centered gene expression for calculating expression scores in Lund and TCGA cohort analyses; noncentered gene expression scores were used in pan‐cancer analysis of correlation between methylation and gene expression.

## 3 Results

### 3.1 DNA methylation‐based immune profiling of metastatic melanoma tumors

**Fig. 1:**
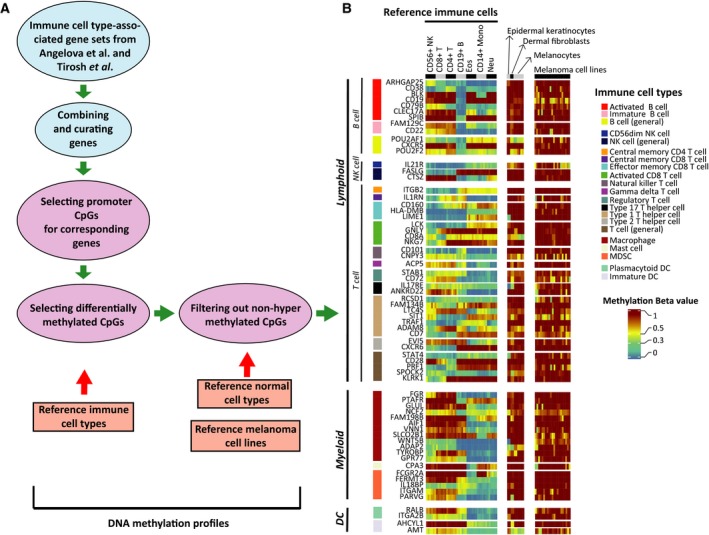
Strategy to identify immune cell type‐specific CpGs. (A) Schematic outline of the strategy to select immune cell type‐associated CpGs by combining Angelova *et al.* (2015) and Tirosh *et al.* (2016) signatures. (B) Heatmap of the methylation profiles for the reference cell types (immune, normal, and melanoma cell lines) along the selected 67 CpGs. Color bars on the left indicate the corresponding cell type for the CpG. Color bar on the right indicates the corresponding range for methylation β. Row names indicate corresponding gene names for the CpGs.

To explore the DNA methylation immune landscape in MM tumors, we first curated two previously described immune gene expression signatures (Angelova *et al.*, 2015; Tirosh *et al.*, 2016) (Fig. 1A) to identify corresponding gene promoter CpGs. Next, we identified those CpGs that were most differentially methylated across seven normal peripheral blood‐derived immune cell subsets (Reinius *et al.*, 2012), while in parallel showing high methylation in nonimmune cell types in the tumor microenvironment (cancer cell lines and normal stromal cells—dermal fibroblasts, epidermal keratinocytes, and melanocytes) (Lauss *et al.*, 2015). After subsequent selection of the best CpG (CpG showing lowest FDR in Fisher's exact test for differential methylation) for individual genes, we arrived at a final list of 67 CpGs (Table S1). These, we hypothesize, are DNA methylation signatures representative of different immunological cell types (Fig. 1B). Next, we used this CpG set to analyze Illumina EPIC methylation array‐based profiles from 180 MM tumors (the Lund cohort, Table S2).

### 3.2 Immune cell type‐associated CpGs reveal melanoma subgroups

**Fig. 2:**
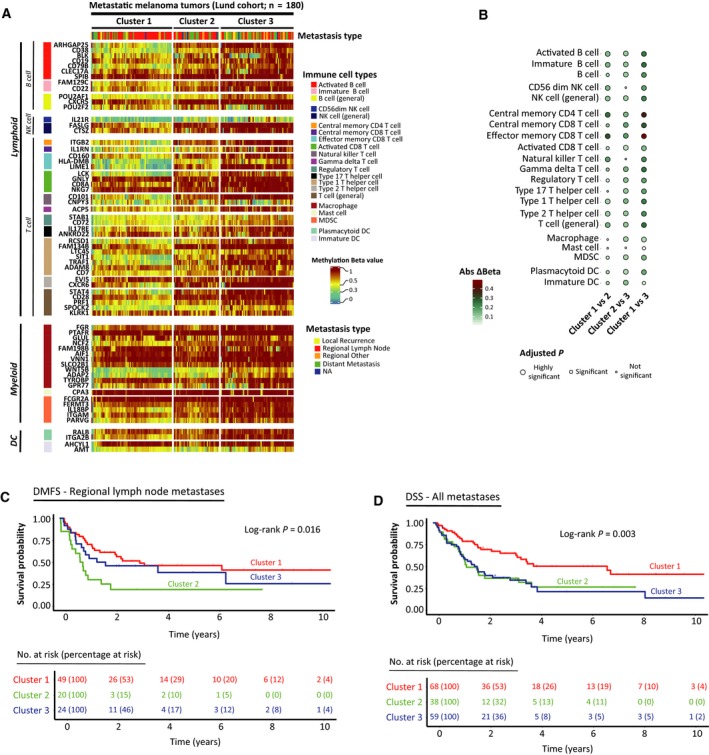
Immune methylation clusters and association with survival. (A) Selected immune cell type‐specific CpGs for immune methylation clusters across Lund cohort. Color bar on the right indicates the corresponding range for methylation β. Color bars on the left indicate the corresponding cell type for the CpG. Row names indicate corresponding gene names for the CpGs. (B) Balloon plot of absolute methylation difference per immune cell type between clusters. Balloon colors are representative of absolute Δβ, and balloon sizes indicate level of significance for comparisons. Color bar on the bottom left indicates the corresponding range for methylation absolute Δβ. *P*‐values calculated using Kruskal–Wallis and *post hoc* Dunn test, and multiple testing corrections performed across cell types for each comparison individually. (C, D) Kaplan–Meier plots of the immune methylation clusters for DMFS (regional lymph node metastases only) and DSS (all metastases except local recurrences), respectively. Numbers at risk and percentages at risk (within brackets) are indicated below each plot. *P*‐values calculated using log‐rank test. Maximum time to display on *x*‐axis has been clipped to 10 years.

Consensus clustering of the Lund cohort using the selected immune cell type‐specific CpGs identified three immune methylation clusters (Fig. S1A). Cluster 1 showed overall lower methylation in the majority of the immune CpGs, whereas Cluster 3 displayed the opposite, with Cluster 2 being intermediate in appearance (Fig. 2A). To explore the observed methylation differences further, we constructed a median methylation score for all CpGs associated with the described immune cell types for each tumor. We found that differences in methylation scores between Clusters 1 and 3 were most pronounced, with 19 out of 21 immune cell types being significant (Fig. 2B, Kruskal–Wallis and *post hoc* Dunn test; multiple testing corrections across cell types for each comparison individually, FDR < 0.001). The biggest difference in methylation values was observed for central memory CD4^+^ T cells (absolute Δβ: 1 vs 2: 0.25; 2 vs 3: 0.14; 1 vs 3: 0.39; FDR < 0.001) and effector memory CD8^+^ T cells (absolute Δβ: 1 vs 2: 0.3; 2 vs 3: 0.18; 1 vs 3: 0.48; FDR < 0.001). Significant differences between Clusters 1 and 2 were observed mostly in the lymphoid lineage and differences between Clusters 2 and 3 followed a similar pattern of significance to that of Clusters 1 and 3, albeit with lower level of methylation difference (absolute Δβ‐value).

### 3.3 Immune methylation clusters are associated with patient survival

To determine whether the identified methylation patterns have prognostic value, we analyzed for differences in the survival outcome of the melanoma patients in Lund cohort. Immune methylation clusters showed significant difference in survival using both distant metastasis‐free survival (DMFS) for regional metastatic cases and melanoma‐specific survival for the entire metastatic cohort (DSS) using univariate Cox regression models (Fig. 2C,D, respectively). Cluster 2 showed significantly decreased DMFS [hazard ratio (HR): 2.45; 95% confidence interval (CI): 1.30–4.61; *P* = 0.005] compared to Cluster 1. Regarding DSS, we found both Cluster 2 (HR: 2.10; 95% CI: 1.22–3.64; *P* = 0.008) and Cluster 3 (HR: 2.18; 95% CI: 1.33–3.56; *P* = 0.002) to be of significantly higher risk when compared to Cluster 1. Furthermore, multivariable Cox regression models with adjustments for age and gender (DMFS) and metastasis type (DSS) reported analogous findings for both DMFS (Fig. S1B) and DSS (Fig. S1C). Overall, using DNA methylation‐based immune profiling we identified three melanoma subgroups with different patient survival.

### 3.4 Molecular characterization of immune methylation clusters

**Fig. 3:**
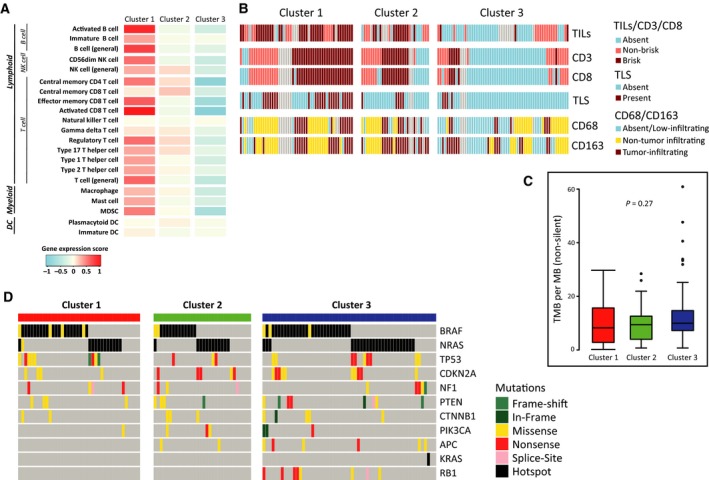
Molecular characteristics of immune methylation clusters. (A) Microarray‐based gene expression score for the selected immune cell types across clusters. Color bar on the bottom indicates the corresponding range for expression score. (B) Distribution of staining categories across clusters for TILs, and CD3^+^ and CD8^+^ T cells along with the information on the presence of TLS in the tumor and similar IHC staining for CD68^+^ and CD163^+^ myeloid cells. Staining categories are represented as follows: absent (absence of infiltration), nonbrisk (localized infiltration), and brisk (Strong infiltration) for TILs, CD3, and CD8; absent/low‐infiltrating (absence or low infiltration), non‐tumor‐infiltrating (infiltration outside tumor boundary), and tumor‐infiltrating (infiltration inside tumor boundary) for CD68 and CD163. (C) Distribution of nonsilent tumor mutational burden (per MB) across clusters. *P*‐value calculated using Kruskal–Wallis test. (D) Oncoplot of important melanoma‐associated mutations across clusters. Color bar on the right indicates the mutation category. Hotspot mutations are defined to involve V600, K601 for BRAF, and Q61, G12, and G13 for RAS.

Next, we set out to explore whether the identified immune methylation clusters were associated with transcriptional differences of the immune cell‐specific genes in matched tumors (*n* = 179). Overall, transcriptional differences followed the observations on DNA methylation levels (Fig. 3A). Indeed, for most selected immune cell CpGs, moderate‐to‐high negative correlations (−0.2 to −0.5 and below, τ_Kendall_) were found between methylation and expression of the corresponding genes, as expected for the promoter CpGs involved in the regulation of gene expression (Fig. S1D). Next, we investigated whether the identified methylation clusters were accompanied by differences in immune cell infiltration using hematoxylin and eosin staining for tumor‐infiltrating lymphocytes (TILs) along with immunohistochemical staining (IHC) for CD3 and CD8 for T cells, and CD68 and CD163 for myeloid cells. Using matched staining data from 127 of 180 MM tumors, we analyzed association between clusters and staining categories (strong infiltration, localized infiltration, and absent for TILs and T cells; absent/low infiltration, nontumor infiltration, and tumor infiltration for myeloid cells). In line with the DNA methylation data, Cluster 1 had the highest percentage of samples with strong and localized infiltration of TILs, CD3^+^, and CD8^+^ T cells (*P* < 0.001; FDR < 0.001) (Fig. 3B). The presence of TILs showed significant association with DSS in the Lund cohort (Fig. S1E). Regarding CD68^+^ and CD163^+^ myeloid cells, Cluster 1 had fewer absent/low‐infiltrating samples compared to Cluster 3. Together, these results support that tumor infiltration of lymphocytes and myeloid cells is accurately reflected by DNA methylation.

Recently, the existence and importance of tertiary lymphoid structures (TLS) in the melanoma tumors have been reported along with their close relationship with prognosis and response to immunotherapeutic treatments (Cabrita *et al.*, 2020; Helmink *et al.*, 2020). Here, using the matched TLS information for the tumors (Cabrita *et al.*, 2020), we observed significant enrichment of TLSs in Cluster 1 tumors (*P* < 0.001; FDR < 0.001) (Fig. 3B), indicating the possibility of a successful antitumor immunity in these tumors.

Tumor mutational burden has emerged as an important biomarker for predicting response to ICI treatments (reviewed in Chan *et al.*, 2019). Using mutation data from 1368 cancer genes in 129 of 180 MM tumors from the Lund cohort, we did not observe a relationship between immune methylation clusters and tumor mutational burden or specific melanoma hotspot mutations (Fig. 3C,D, respectively). Additionally, we analyzed association between immune methylation clusters and our previously reported gene expression phenotypes (Cirenajwis *et al.*, 2015) and observed strong association between high‐immune class and low methylation Cluster 1 (Fisher's exact *P* < 0.001, Table S3).

Overall, the immune methylation clusters are independent of mutational patterns and tumor mutational burden but are confirmed by the presence or absence of immune cells on the mRNA and protein level.

### 3.5 Analysis of immune exclusion mechanism associated with immune methylation clusters

**Fig. 4:**
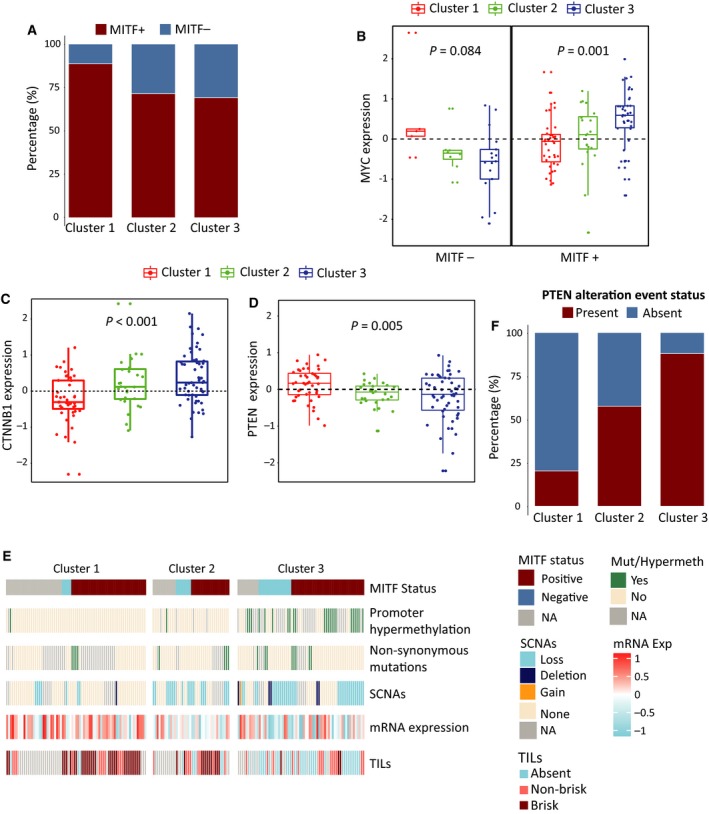
Exploration of immune exclusion mechanisms in the Lund clusters. (A) Barplot showing the distribution of MITF^+^ and MITF^−^ samples across immune methylation clusters. (B) Boxplot showing distribution of c‐MYC (*MYC*) mRNA expression for MITF^−^ samples across clusters (left) and same for MITF^+^ samples (right). *P*‐value from Kruskal–Wallis test. (C, D) Boxplots showing distribution of β‐catenin (*CTNNB1*) and *PTEN* mRNA expression across clusters, respectively. *P*‐value from Kruskal–Wallis test. (E) Heatmap showing distribution of different molecular alteration events in *PTEN* along with the corresponding gene expression for samples across clusters and staining information for TILs, further stratified by sample MITF status. Staining categories are represented as follows: absent (absence of infiltration), nonbrisk (localized infiltration), and brisk (Strong infiltration) for TILs. (F) Barplot showing distribution of overall *PTEN* alteration event status across clusters. A PTEN alteration event is defined as either promoter hypermethylation, nonsynonymous mutation, or copy number loss/deletion.

Several molecular mechanisms have been proposed to explain why certain tumors are able to escape the immune system (Casey *et al.*, 2016; Gettinger *et al.*, 2017; Le *et al.*, 2017a; Peng *et al.*, 2016; Spranger *et al.*, 2015; further reviewed in Spranger and Gajewski, 2018; Trujillo *et al.* 2018). Clearly, immune methylation Cluster 3 was characterized by an immune‐poor DNA methylation pattern reflected by increased methylation levels of immune cell‐specific CpGs (Fig. 2A). Further, we hypothesized that the differentiation state of the melanoma tumor may determine the ability of Cluster 3 tumors to evade the immune system. Thus, we used matched staining information for 127 of 180 MM tumors for the MITF protein, which is a melanocyte‐specific transcription factor important in melanocyte differentiation and melanoma cell survival (Carreira *et al.*, 2005; Garraway *et al.*, 2005; McGill *et al.*, 2002; reviewed in Levy *et al.*, 2006; Steingrímsson *et al.*, 2004). Overall, 25% of the matched stained tumors were negative for MITF protein. Intriguingly, we found fewer MITF‐negative tumors in the immune‐rich methylation Cluster 1 (Fig. 4A; chi‐square *P* = 0.06). As MITF‐positive and MITF‐negative tumors are known to harbor different transcriptional programs and presumably represent two distinct melanoma states (Hoek *et al.*, 2006), we thus performed subsequent analyses in MITF‐positive and MITF‐negative tumors separately. In pathway analysis of gene expression data, we found that MITF‐positive tumors showed enrichment for c‐MYC (*MYC*) target genes and DNA repair‐associated genes in Cluster 3 (FDR < 0.05); however, such observations were not found for the MITF‐negative group. Upon stratification by MITF status, we observed a significant difference in *MYC* expression across clusters for MITF‐positive tumors (Fig. 4B, *P* = 0.001), which was not observed for the combined data (Fig. S2A). Furthermore, we explored the molecular profiles of known immune exclusion‐associated genes (beta‐catenin pathway, PI3K pathway, *TP53*, reviewed in Spranger and Gajewski (2018)) across clusters. *CTNNB1* showed a significant mRNA expression difference across clusters (Fig. 4C, *P* < 0.001, FDR < 0.05); however, when stratified by MITF status, characteristics similar to *MYC* were revealed (Fig. S2B, *P* < 0.001). Together, these observations delineate that *MYC* and *CTNNB1* may act as immune exclusion molecules in MITF‐positive melanoma tumors. Among PI3K‐Akt pathway genes, we observed significant gene expression differences across clusters for *PTEN* and *PIK3R1* (Fig. 4D and Fig. S2C, *P* = 0.005; FDR < 0.05 and *P* = 0.03; FDR < 0.05, respectively) irrespective of MITF status. Upon further investigation, we found that *PTEN* somatic copy number alterations (SCNAs) showed good overlap with *PTEN* promoter hypermethylation and nonsynonymous mutations for the same tumors. However, *PTEN* hypermethylation and mutations were found to be mostly exclusive (Fig. 4E, Fig. S2D). Next, we summed up all molecular alteration events of *PTEN* for each tumor and found significant concordance between *PTEN* alteration events and corresponding mRNA expression (Fig. S2E). Also, the distribution of tumors harboring any *PTEN* alteration event varied significantly across clusters (chi‐square *P* < 0.001) with Cluster 3 showing highest enrichment (Fig. 4F). We further explored the association between *PTEN* mRNA expression and TILs and observed a trend in difference in terms of *PTEN* expression between tumors with absent and brisk TILs (Fig. S2F). The association of TILs with molecular events in *PTEN* was more prominent (Fig. 4E), and a significant difference in terms of TILs was observed between tumors with and without PTEN molecular events (chi‐square *P* = 0.001). Association between immune methylation clusters and molecular event status in PTEN was also validated in the TCGA MM cohort (Fig. S2G). Together, we observed a known immune exclusion mechanism in the immune‐poor MM tumors displaying a complex pattern with upregulation of PI3K‐Akt pathway by blocking *PTEN* functioning through molecular alterations and upregulation of *MYC* and *CTNNB1* in MITF‐positive tumors only.

### 3.6 Validation of immune methylation clusters in external melanoma cohorts

**Fig. 5:**
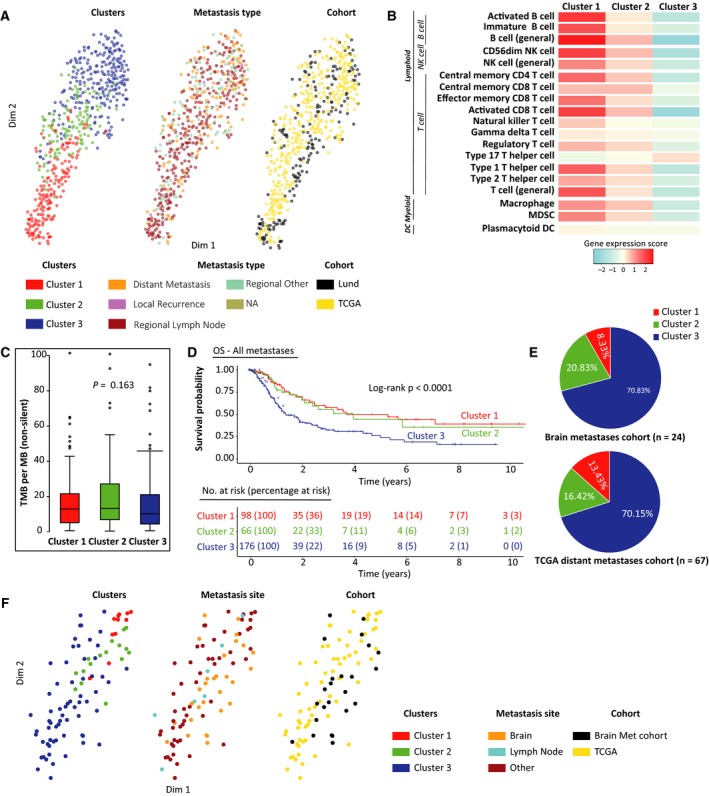
Validation of immune methylation clusters in external melanoma cohorts. (A) tSNE plot of methylation profiles for Lund and TCGA tumors using common centroid CpGs, annotated by clusters (left), metastasis type (site) (middle), and cohort membership (right). (B) RNA‐Seq‐based gene expression scores for the selected immune cell types across TCGA clusters. Color bar on the bottom indicates the corresponding range for expression score. (C) Distribution of nonsilent tumor mutational burden (per MB) across TCGA clusters. *P*‐value calculated using Kruskal–Wallis test. (D) Kaplan–Meier plot for the TCGA clusters for OS. Numbers at risk and percentages at risk (within brackets) are indicated below the plot. *P*‐value calculated using log‐rank test. Maximum time to display on *x*‐axis has been clipped to 10 years. (E) Pie charts of cluster membership distribution for brain metastases cohort (top) and TCGA distant metastases (bottom). (F) *tSNE* plot of methylation profiles for TCGA distant metastatic tumors and melanoma brain metastases from the brain metastasis cohort using common centroid CpGs, annotated by cluster membership (left), metastasis site (middle), and cohort membership (right).

To confirm the immune methylation clusters, we built methylation‐based centroids to classify samples belonging to the TCGA MM cohort. For this, we selected CpGs from the immune CpG set (Table S1) which showed a significant methylation difference across Lund clusters (Kruskal–Wallis test, FDR < 0.0001). Median methylation values for these significant CpGs (*n* = 51) were computed for each cluster to form methylation centroids (Table S4). A minor fraction of samples (2.37%) could not be classified (τ_Kendall_ < 0.3). Immune methylation clusters in TCGA cohort displayed methylation profiles similar to corresponding clusters in the Lund cohort (Fig. S3A). When we grouped both the Lund and TCGA cohorts together using common centroid CpGs and *tSNE* (van der Maaten and Hinton, 2008) (perplexity = 30; θ = 0.1), we noticed clear segregation between the immune methylation clusters in the first two dimensions, as expected (Fig. 5A, left). Such segregation was not observed when samples were annotated on the basis of the site of metastasis (metastasis type) or cohort (Fig. 5A middle and right, respectively). Additionally, gene expression scores, tumor mutational burden, and mutational patterns displayed identical characteristics for clusters as observed in the Lund cohort (Fig. 5B,C, Fig. S3B, respectively). Importantly, Cluster 3 displayed a significant inferior overall survival (OS, HR: 2.29; 95% CI: 1.54–3.40; *P* < 0.001) compared to Cluster 1 in TCGA data (Fig. 5D) and remained significant in multivariate analyses with adjustment for metastasis type (*P* < 0.001, Fig. S3C). Additionally, we analyzed the association between immune methylation clusters in TCGA cohorts and previously reported gene expression classes for the same cohort (Akbani *et al.*, 2015) and observed strong association between Immune class and low methylation Cluster 1 (Fisher's exact *P* < 0.001, Table S5).

To further explore immune gene methylation in distant metastases, we classified brain, lymph node, and other metastases from distant sites from TCGA skin cutaneous melanoma (SKCM) cohort (*n* = 68) and brain metastases (*n* = 30) from another cohort (http://www.ncbi.nlm.nih.gov/geo/query/acc.cgi?acc=GSE108576) (Orozco *et al.*, 2018) into the immune methylation clusters using methylation‐based centroids. We found that samples in both cohorts were enriched for Cluster 3 irrespective of the metastasis location (Fig. 5E). In TCGA cohort, we did not find any significant association between metastasis location and cluster membership. When both cohorts were analyzed together across the common centroid CpGs and *tSNE* (perplexity 30; θ = 0.1), observations similar to earlier Lund and TCGA joint analysis were made (Fig. 5F). Together, the immune cell methylation characteristics of the Lund cohort were re‐identified in external data, with similar prognostic implications, and the immune methylation profiles were largely independent of the site of metastasis.

### 3.7 Implications of the epigenetic immune clusters in a pan‐cancer context

**Fig. 6:**
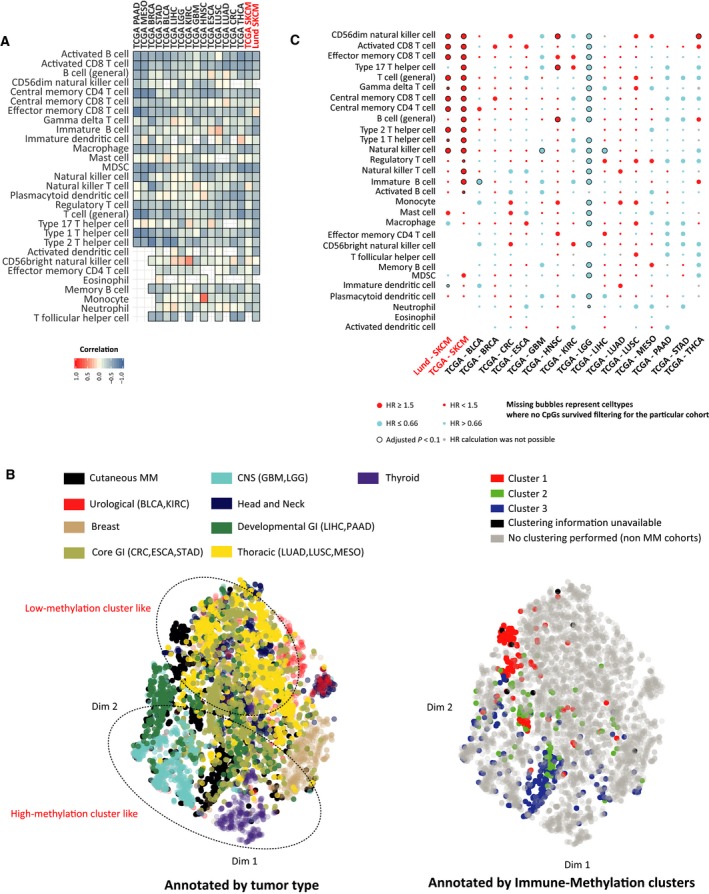
Immune‐specific methylation in pan‐cancer context. (A) Correlation between tissue‐specific immune cell type methylation and corresponding gene expression scores for MM cohorts (Lund and TCGA SKCM) and 15 other TCGA primary cancer cohorts (BLCA, bladder urothelial carcinoma; BRCA, breast invasive carcinoma; CRC, colorectal carcinoma; ESCA, esophageal carcinoma; GBM, glioblastoma multiforme; HNSC, head and neck squamous cell carcinoma; KIRC, kidney renal clear cell carcinoma; LGG, lower grade glioma; LIHC, liver hepatocellular carcinoma; LUAD, lung adenocarcinoma; LUSC, lung squamous cell carcinoma; MESO, mesothelioma of lung; PAAD, pancreatic adenocarcinoma; STAD, stomach adenocarcinoma; THCA, thyroid carcinoma). MM cohorts are highlighted in red. Methylation scores for MM cohorts were calculated from the melanoma‐specific 67 immune CpG set, whereas for other tumor types, they were calculated using individual tissue type‐specific immune CpG sets in the similar fashion to MM. (B) *tSNE* plot of pan‐cancer cohorts based on the previously mentioned methylation scores of common 17 immune cell types; plot is annotated by tissue of origin (left) and immune methylation cluster membership for MM (right). (C) Balloon plot of hazard ratios (HR) for dichotomized immune methylation scores (β > 0.7: hypermethylated, β ≤ 0.7: not hypermethylated) from univariate Cox regression models for each immune cell type and cancer cohorts. MM cohorts are highlighted in red. Balloon colors are indicative of direction of hazard ratios, and sizes indicate value category of HR (large: HR ≥ 1.5 or HR ≤ 0.66; small: HR < 1.5 or HR > 0.66). Additional black circles indicate significance after correction for multiple testing (Benjamini–Hochberg corrected *P*‐value < 0.1). Gray circles indicate situations where HR calculations were not possible, and missing circles indicate cell types where no CpG survived filtering.

Next, we wanted to investigate whether immune methylation signatures have the same prognostic implications in other cancer types as in melanoma, and to which extent immune environments display shared characteristics among solid tumors. At first, we wanted to dissect the role of specific immune cell lineages across cancer types. To this purpose, we created immune cell methylation scores for MM tumors (both Lund and TCGA cohorts) using previously derived melanoma‐specific immune CpG set. Similar scores for non‐MM TCGA solid tumor cohorts were constructed by identifying immune cell type‐specific CpGs individually for each cohort in a process analogous to MM. Here, the immune CpGs were filtered against cancer type‐specific CpGs using cell lines from the Genomics of Drug Sensitivity (GDSC) database. The resulting methylation scores were highly anticorrelated to the corresponding gene expression scores (computed in the similar manner as methylation scores, using genes instead of the corresponding CpGs), for most immune cell types and tumor cohorts (Fig. 6A). Next, to explore the relations among the immune microenvironments across tumor types, we constructed a *tSNE* plot using aforementioned methylation scores for 17 immune cell types. When tumor types were annotated on the basis of their tissue of origin, we observed some tumor cohorts were more localized than others (Fig. 6B, Left). Known immune‐deprived tumor types like CNS tumors (lower grade glioma, LGG; and glioblastoma multiforme, GBM) colocalized with a group of similarly immune‐poor tumor types from the developmental gastrointestinal tumors (pancreatic adenocarcinoma, PAAD; and liver hepatocellular carcinoma, LIHC), thus indicating that immune microenvironments of these tumor types bear resemblance despite their diverse tissue of origin. Moreover, the immune methylation Clusters 1 and 3 from MM separated well in this immune microenvironment plot (Fig. 6B, Right). Cluster 1 tumors were found to be colocalizing with a group of diverse tumor types, indicating existence of similar type of immunologically active tumors across the cancer spectrum. Conversely, we found Cluster 3 tumors colocalizing with most CNS and endocrine tumors, indicating that the immune microenvironment of these immune‐poor melanomas likely resembles other immune‐poor tumor types. Next, we analyzed the prognostic implications of immune cell type methylation scores across the cohorts using overall patient survival (OS). For this, we dichotomized the methylation scores into two categories, β > 0.7 as hypermethylated and β ≤ 0.7 as not hypermethylated. We found methylation scores for CD56^+^ dim NK cells, and activated and effector memory CD8^+^ T cells, to be significantly prognostic across multiple cancer cohorts including melanoma, with low methylation levels inferring good prognosis in most cancer types (Fig. 6C). Overall, we found low methylation scores of immune cell types to be associated with good outcome for the majority of cell types, particularly for immune cells from the lymphoid lineage, which might reflect the role of the adaptive immune system for an effective response to tumor neoantigens. However, the majority of significant associations with good outcome (adjusted *P* < 0.1) occurred in only two tumor types, that is, MM and head and neck squamous cell carcinoma (HNSC). Collectively, methylation‐based immune cell signatures inform on prognosis in several solid tumor cohorts, particularly in melanoma.

## 4 Discussion

In this study, we report that immune methylation‐specific signatures harbor important prognostic information and simultaneously inform us about the different tumor immune microenvironments across cancer landscape with a particular focus on MM. DNA methylation has established its role as a major epigenetic driver in cancer progression and development. However, its contribution to define the characteristics of tumor microenvironment remains poorly understood. It has been pointed out recently that DNA hypomethylation promotes immune evasion in the corresponding tumors (Jung *et al.*, 2019). Also, DNA methylation pattern predicting response to ICI treatment for non‐small‐cell lung cancer has been unveiled (Duruisseaux *et al.*, 2018). Thus, further emphasis on DNA methylation for the distinction of different tumor microenvironments is warranted. Our analyses with an immune cell type‐specific CpG set have unraveled diversity in the immune landscape of MM tumors in terms of DNA methylation. We identified three immune methylation clusters that were significantly associated with patient survival and further supported by transcriptomic and immunostaining data. Together, these analyses have hinted at the inverse relationship between immune cell type‐specific methylation and enrichment of the corresponding immune cell types in the tumor microenvironment.

Tumor mutational burden has often been associated with positive clinical response to ICI treatments for several tumor types including melanoma (Goodman *et al.*, 2017; Van Allen *et al.*, 2015, reviewed in Chan *et al.*, 2019). However, we did not observe a clear association of immune methylation clusters with the tumor mutational burden, in line with a previous report (Spranger *et al.*, 2016). This might be due to a universally high immunogenicity of melanoma cells, where immune evasion, as observed in Cluster 3, that is, immune methylation high cluster, can rather be achieved by transcriptional changes (Jerby‐Arnon *et al.*, 2018). Therefore, in this study we additionally explored the possible immune exclusion mechanisms (reviewed in Spranger and Gajewski, 2018) at play for the Cluster 3 tumors in our cohort. Upregulation of PI3K‐Akt pathway and subsequent downregulation of *PTEN* are a major immune evasion mechanism that has been observed across cancers including melanoma (reviewed in Dituri *et al.*, 2011; Spranger and Gajewski, 2018). However, somatic mutations in *PTEN* gene have found to be infrequent in melanomas (reviewed in Aguissa‐Touré and Li, 2012); hence, alternative molecular mechanisms such as SCNAs and promoter hypermethylation have been proposed as an alternative mechanism of blocking *PTEN* functions (Roh *et al.*, 2016; Stahl *et al.*, 2003). Our observations for Cluster 3 tumors further supported the hypothesis that immune‐poor melanomas likely achieve immune evasion through blocking of PTEN functioning through promoter hypermethylation and SCNAs and often in conjunction with one another. The role of *MITF* as a major transcription factor regulating melanoma progression and development is well established (Carreira *et al.*, 2005; Garraway *et al.*, 2005; McGill *et al.*, 2002; reviewed in Levy *et al.*, 2006; Steingrímsson *et al.*, 2004). *MITF*‐low melanomas have been shown to be resistant to multiple targeted treatments (Müller *et al.*, 2014). Studies have also shown that decreased expression of melanocyte differentiation antigens through downregulation of *MITF* could possibly trigger immune evasion in melanomas (Kono *et al.*, 2006; Woods *et al.*, 2014). Nonetheless, exact role of *MITF* in the immune exclusion mechanism is still unclear. Our analyses with immune‐poor melanomas stratified by MITF immunostaining suggested transcriptional upregulation of β‐catenin‐dependent canonical Wnt signaling pathway along with the upregulation of c‐Myc in the MITF‐positive group. Immune evasion through upregulation of canonical Wnt signaling pathway is common in melanomas (Spranger *et al.*, 2015), and the role of c‐Myc in the immune exclusion process has previously been described (reviewed in Casey *et al.*, 2018). Since β‐catenin‐induced melanomas require functional *MITF* (Schepsky *et al.*, 2006; Widlund *et al.*, 2002) and loss of *MITF* expression affects the corresponding expression of c‐Myc (Seoane *et al.*, 2019), thus the role of *MITF* seems important in distinction of the immune evasion mechanisms in melanoma. As immune‐low tumors respond poorly to immunotherapeutic treatments (Cristescu *et al.*, 2018; Van Allen *et al.*, 2015), hence these results suggest further studies to clarify the role of *PTEN* and *MITF* levels in immune exclusion.

Notably, our analyses on distant MMs did not reveal a major difference in the immune microenvironment characterization among tumors from different locations, most notably brain. This is further supported by clinical trials showing efficacy of ICI for intracranial metastases concordant with the extracranial ones and systemic response (Kluger *et al.*, 2019; Margolin *et al.*, 2012; Tawbi *et al.*, 2018). A recent study using mouse models suggested such efficacy is likely due to infiltration of T cells especially CD44^+^CD62L^−^ effector memory cells from the extracranial sites (Taggart *et al.*, 2018).

Our analyses on a pan‐cancer cohort revealed similarities in the immune microenvironments of tumors from diverse tumor types in terms of immune methylation. Generally, our findings are compatible with the concept of inflammatory and noninflammatory tumor environments (Chakravarthy *et al.*, 2018); however, our pan‐cancer analyses suggest that immune environments carry additional complexity beyond this concept, as has been demonstrated before (Thorsson *et al.*, 2018). Using cohort‐specific immune methylation scores, we observed grouping of MM tumors belonging to the low immune methylation cluster along with tumors from lung and gastrointestinal (GI) tract. Such observation indicates that immune‐rich microenvironments from melanoma have similarities to microenvironment of lung and GI tumors. In this regard, it is interesting that ICI treatments have garnered significant attention for non‐small‐cell lung cancer (NSCLC), which is among the cancer types where such an immune‐rich microenvironment likely occurs (reviewed in Moon *et al.*, 2017). Therefore, in addition to a high mutational burden, such as induced by microsatellite instability (MSI) (Le *et al.*, 2015), an immune‐rich environment may confer improved outcome to immunotherapy agents. Among the different immune cell types, the prognostic value of effector memory T cells could also be established in several tumor types of the pan‐cancer cohort. Memory T cells have received attention recently, as their presence in the tumor microenvironment was associated with clinical response to ICI treatment in melanoma (Gide *et al.*, 2019; Sade‐Feldman *et al.*, 2018).

## 5 Conclusion

In summary, we explored the immune microenvironment in MM tumors from a DNA methylation perspective. DNA methylation offers several advantages over transcriptomic characterization of mixed cellular environments, primarily due to its higher frequency in terms of differences between cellular lineages compared to gene expression (Bock *et al.*, 2012). Additionally, the biologically limited range of methylation β‐values, being restricted from 0 (unmethylated) to 1 (methylated), facilitates the comparison across experiments and laboratories, whereas transcriptomic data naturally have a higher dynamic range. Also, greater stability of DNA compared to RNA in formalin‐fixed, paraffin‐embedded (FFPE) samples (Okello *et al.*, 2010) makes it easier to work with DNA methylation in archival, historical cohorts.

Our use of a largely untreated historic cohort ensured that the immune cell type methylation patterns are not biased by treatment. Also, the extension of our study to other solid tumors revealed an immune‐rich environment in several other cancer types. The prognostic implications of some innate and adaptive immune cell types were re‐identified in other cancer types. These results point toward a more tailored approach to immunotherapy based on tumor immune microenvironment.

## Conflict of interest

The authors declare no conflict of interest.

## Author contributions

GJ and KMB conceived the project and received appropriate funding; GJ, ML, and SM designed the study with inputs from JS and MR; KMB, JC, and TZ performed Illumina EPIC array hybridization and initial preprocessing of the data; KI, HO, CI, AC, and KN provided patient tumor materials; SM performed bioinformatic analyses with inputs from ML, GJ, RC, JS, and MR; RC performed IHC analyses; SM, ML, and GJ contributed to the manuscript writing with inputs from KMB, JS, and MR. All authors approved the final draft of the manuscript.

## Supporting information


**Fig S1.** Additional characteristics of Lund immune‐methylation clusters.

**Fig S2.** Additional characteristics of immune exclusion in the immune‐methylation clusters.

**Fig S3.** Additional characteristics of TCGA immune‐methylation clusters.

**Table S1.** List of immune cell type associated CpGs with corresponding gene and associated cell type information.
**Table S2.** Clinical characteristics for immune‐methylation clusters (Lund cohort).
**Table S3.** Overlap between Lund gene expression phenotypes and Lund immune‐methylation clusters.
**Table S4.** List of centroid CpGs with methylation β values for each cluster.
**Table S5.** Overlap between TCGA gene expression classes and TCGA immune‐methylation clusters.
**Appendix S1.** Supplementary methods.
