# Supplementary figures and images for: Analysis of DNA methylation patterns in the tumor immune microenvironment of metastatic melanoma
Source: Mol Oncol. 2020 Mar 21;14(5):933–50. doi: 10.1002/1878-0261.12663 (PMC7191190; doi:10.1002/1878-0261.12663)

Figure S1

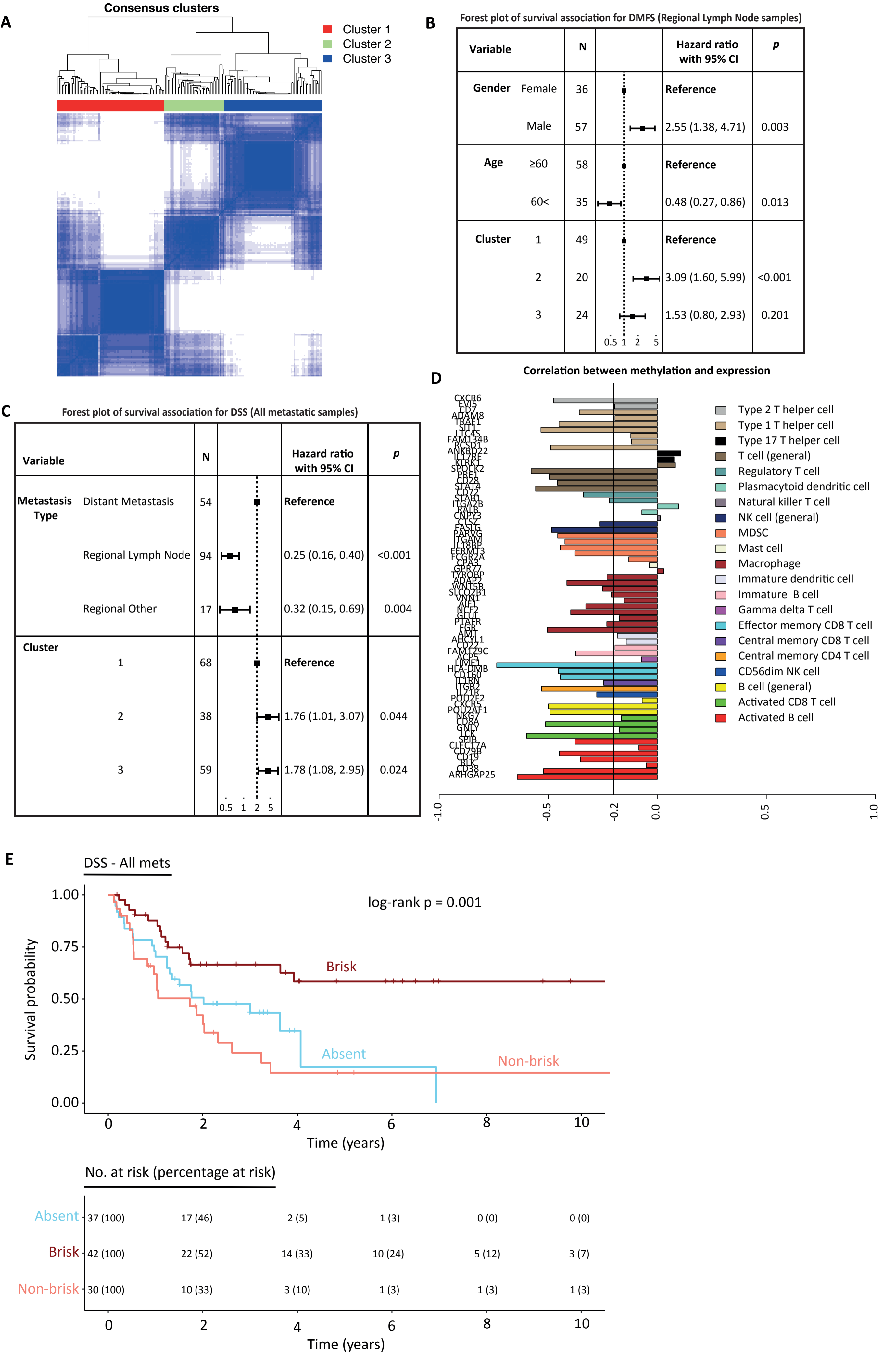

Supplement: Supplementary file 1 — Fig S1. Additional characteristics of Lund immune‐methylation clusters. [file MOL2-14-933-s001.pdf]

**Figure S2**

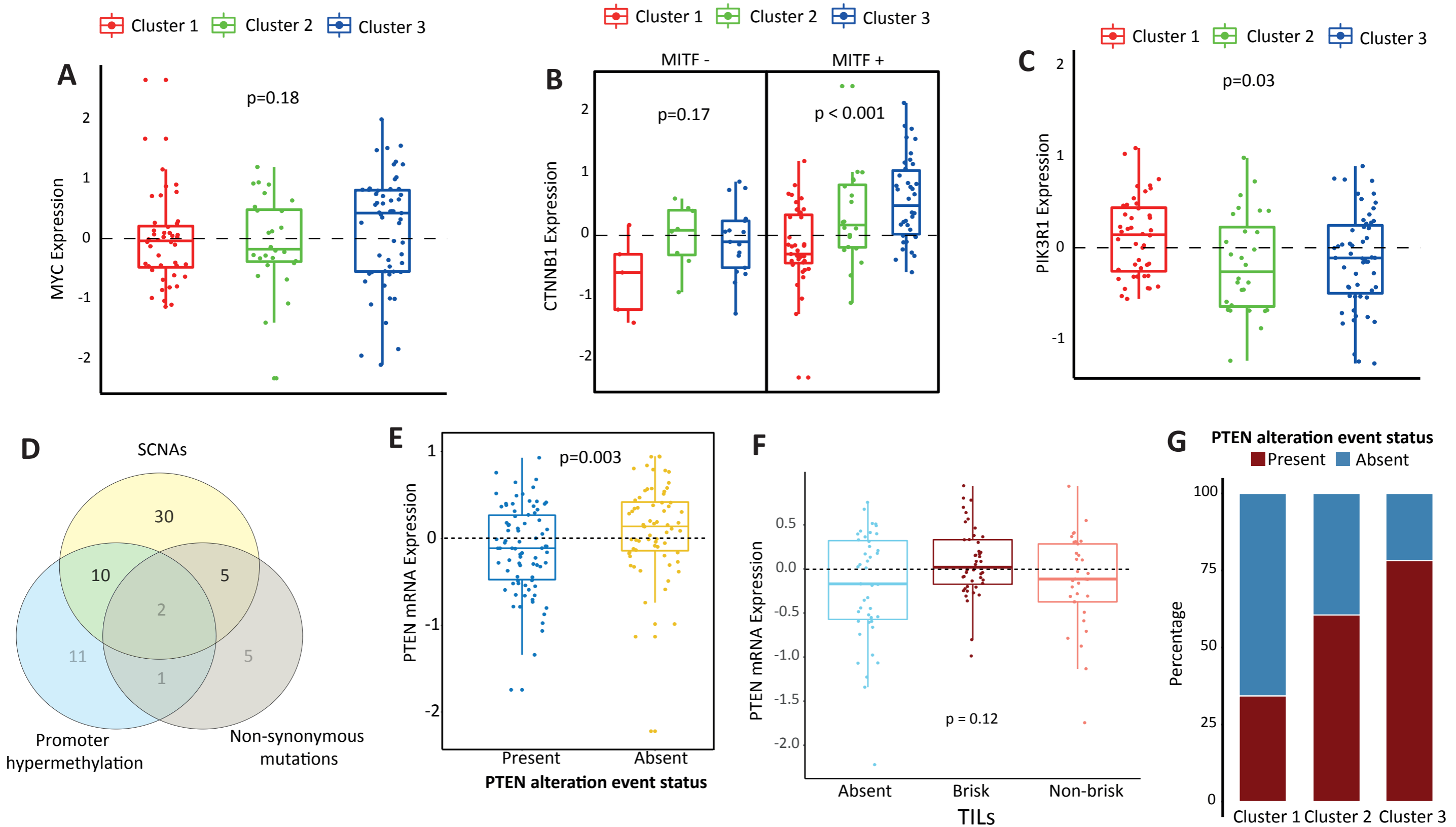

Supplement: Supplementary file 2 — Fig S2. Additional characteristics of immune exclusion in the immune‐methylation clusters. [file MOL2-14-933-s002.pdf]
