# Supplementary material for: Analysis of DNA methylation patterns in the tumor immune microenvironment of metastatic melanoma
Source: Mol Oncol. 2020 Mar 21;14(5):933–50. doi: 10.1002/1878-0261.12663 (PMC7191190; doi:10.1002/1878-0261.12663)
Supplement: Supplementary file 3 — Fig S3. Additional characteristics of TCGA immune‐methylation clusters. [file MOL2-14-933-s003.pdf]

Figure S3

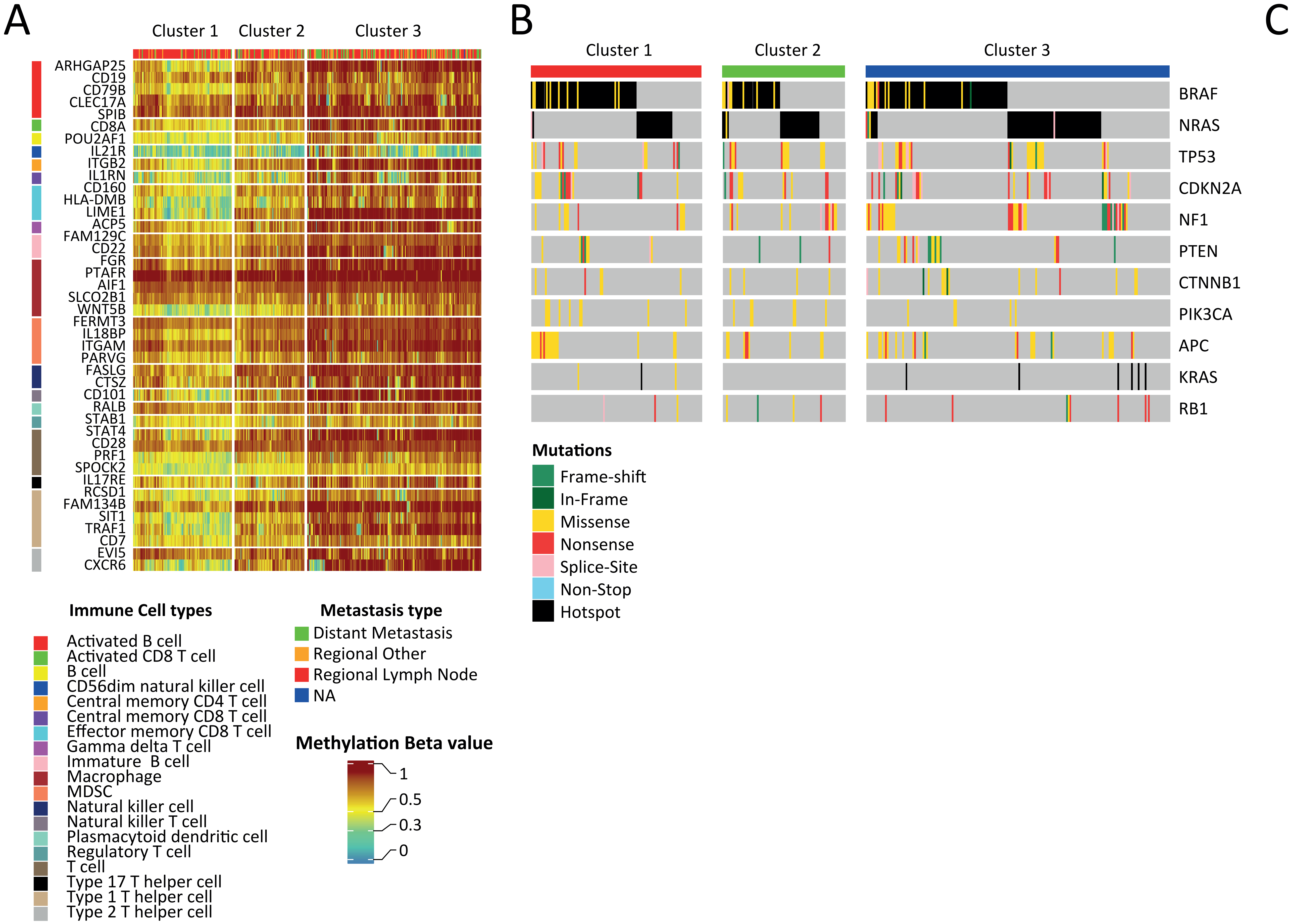

C

Variable	N		Hazard ratio with 95% CI	*p*
**Metastasis type**				
Distant Metastasis	63		**Reference**	
Regional Lymph Node	207		0.67 (0.45, 1.00)	0.05
Regional Other	70		0.55 (0.33, 0.93)	0.03
**Cluster**				
1	98		**Reference**	
2	66		1.17 (0.66, 2.06)	0.59
3	176		2.15 (1.42, 3.25)	<0.001
		0.5 1 2 3		
