# Supplementary material for: Analysis of DNA methylation patterns in the tumor immune microenvironment of metastatic melanoma
Source: Mol Oncol. 2020 Mar 21;14(5):933–50. doi: 10.1002/1878-0261.12663 (PMC7191190; doi:10.1002/1878-0261.12663)
Supplement: Supplementary file 4 — Table S1. List of immune cell type associated CpGs with corresponding gene and associated cell type information. Table S2. Clinical characteristics for immune‐methylation clusters (Lund cohort). Table S3. Overlap between Lund gene expression phenotypes and Lund immune‐methylation clusters. Table S4. List of centroid CpGs with methylation β values for each cluster. Table S5. Overlap between TCGA gene expression classes and TCGA immune‐methylation clusters. Appendix S1. Supplementary methods. [file MOL2-14-933-s004.docx]

**Supplementary Information**

**Analysis of DNA methylation-based tumor immune microenvironment patterns in metastatic melanoma**

Shamik Mitra^1^, Martin Lauss^1^, Rita Cabrita^1^, Jiyeon Choi^2^, Tongwu Zhang^2^, Karolin Isaksson^3^, Håkan Olsson^1^, Christian Ingvar^3^, Ana Carneiro^4^, Johan Staaf^1^, Markus Ringnér^5^, Kari Nielsen^6^, Kevin M. Brown^2^ and Göran Jönsson^1^

1. Division of Oncology and Pathology, Department of Clinical Sciences, Faculty of Medicine, Lund University, Sweden

2. National Cancer Institute, Division of Cancer Epidemiology and Genetics, US

3. Department of Surgery, Skåne University Hospital, Sweden

4. Department of Oncology, Skåne University Hospital, Sweden

5. Department of Biology, National Bioinformatics Infrastructure Sweden, Science for Life Laboratory, Lund University, 22362 Lund, Sweden

6. Department of Dermatology, Helsingborg General Hospital, Sweden

**Supplementary Methods:**

*Methylation data processing:*

Raw idat files were processed using R(R Core Team 2016) package ChAMP (Morris et al. 2014) and ssNoob (Fortin et al. 2017, Triche et al. 2013) was used as background correction method from minfi (Aryee et al. 2014). Background corrected data was then normalized for type I/II probe using BMIQ (Teschendorff et al. 2013) and filtered for polymorphic and off-target probes (McCartney et al. 2016). Prior to the aforementioned preprocessing, quality control analyses were carried out and 11 samples that failed the QC tests were subsequently removed. Also, one batch of samples showed high technical variation and consisted of too few samples to be adjusted. Hence, all samples from this batch were removed. Additionally, samples with a large number of failed probes (probe detection-p > 0.01; Sample cut-off: >4% of total probes) were removed and probes that failed in more than 10% of the remaining samples were also removed. Next, methylation β values of probes failed in 10% or less samples, were imputed using the *impute.knn* function in R and default settings. After preprocessing, the methylation dataset contained 788,174 probes and 196 samples. The processed methylation data for Lund cohort is available in the Gene Expression Omnibus (GEO) database (<http://www.ncbi.nlm.nih.gov/geo/>), under accession number GSE144487. Additionally, we processed DNA methylation profiles of 14 MITF-low melanoma cell lines using Illumina Infinium MethylationEPIC BeadChip array in the same way, except any probe above the p value cutoff in one or more samples was removed subsequently instead of imputing their methylation β value.

*Other external methylation datasets:*

llumina Infinium 450K Human Methylation BeadChip array data for reference immune cells were obtained from Gene Expression Omnibus (GEO) as GSE35069 (Reinius et al. 2012) and for dermal fibroblasts, epidermal keratinocytes, melanocytes and melanoma cell lines were obtained as GSE51547 (Lauss et al. 2015). DNA methylation data for TCGA tumors were downloaded from the TCGA PanCanAtlas Publications (<https://gdc.cancer.gov/about-data/publications/pancanatlas>). Melanoma brain metastasis methylation profiles were obtained as GSE108576 (Orozco et al. 2018). Cancer cell lines methylation data corresponding to TCGA primary cohorts were downloaded from GDSC (

[https://www.cancerrxgene.org/).](https://www.cancerrxgene.org/))

*Gene expression ,somatic mutations and copy number datasets:*

Illumina Human-HT12v4.0 BeadChip array based gene expression data for Lund tumors were available from an earlier study as GSE65904 (Cirenajwis et al. 2015).

RNA sequencing based gene expression data for TCGA tumors were obtained from TCGA PanCanAtlas Publications and were quantile-normalized and subsequently log transformed as log_2_(data+1).

Somatic mutation profiles for Lund tumors were obtained previously by performing targeted deep sequencing of 1697 cancer associated genes in 146 patients (having a matched blood sample), as previously described (Harbst et al. 2014). Somatic mutations profiles for TCGA SKCM tumors was obtained as mentioned previously (Cirenajwis et al. 2015). Somatic copy number alterations data was obtained from the targeted sequencing data of 1697 genes. Log ratios were generated from bam files of tumor-normal pairs using CONTRA 2.03 with default parameters (Li et al. 2012). Exons with insufficient coverage in the normal sample were removed. Copy number data were segmented using GLAD (Barillot et al. 2004). Somatic copy number alterations data for TCGA SKCM tumors were downloaded from TCGA FireBrowse ([www.firebrowse.org](http://www.firebrowse.org)). LRR(log-ratios) values were converted into four categories, amplification: LRR≥0.9; gain: 0.3≤LRR<0.9; loss: -0.9<LRR≤ -0.3; deletion: LRR≤ -0.9. For calculating overall PTEN events, we only retained loss/deletion events as PTEN SCNA events.

*Immunohistochemistry:*

Tissue microarrays (TMA) were constructed using on average of three 1 mm cores per tumor in an attempt to obtain a representative picture of the tumor. The tissue block was cut in four micrometer sections and then dried at 60^o^ C for one hour. The paraffin embedded sections were deparaffinized and pretreated in the PT-Link (DAKO) with TRS (Target Retrieval Solution)-buffer pH 9. The following steps (except for the primary antibody stainings) were performed in the DAKO staining equipment (Autostainer plus) with Dako kit K8010 solutions: peroxidase block (5 minutes), EnVisionHRP-conjugated polymers (30 minutes), DAB substrate-chromogen solution (2 x 5 minutes), counterstaining with haematoxylin (4 minutes). Between each step, the sections were rinsed with washing buffer. Finally, the sections were dehydrated and mounted with PERTEX mounting media (Ref. 00811) (Histolab). The primary antibodies used were all from Agilent/Dako: CD3 (A0452) in 1:200 dilution, CD8 (M7103) in 1:100 dilution, MITF (Clone C5) in 1:400 dilution, and CD68 (M0718) in 1:1500 dilution. CD163 was used from Novocastra (clone 10D6, product NCL-CD163) in 1:250 dilution. Tumor infiltrating lymphocytes (TIL) were classified using the hematoxylin and eosin staining into absent, non-brisk (clustered) and brisk (tumor infiltrative).

*Consensus Clustering of Lund melanoma tumors:*

Tumors from Lund cohort were clustered using the selected CpG set and applying *consensus clustering* (Monti et al. 2003) function from the R package “ConsensusClusterPlus”. For clustering we categorized methylation β values into 3 numerical categories, 0: β<0.3; 1: β≥0.3 & β≤0.7; 2: β>0.7. Clustering was performed using the following parameters, rep=1000, pItem and pFeature=0.8, clusterAlg=”pam” and distance=”Euclidean”.

**References**

[1] Aryee, M. J., A. E. Jaffe, H. Corrada-Bravo, C. Ladd-Acosta, A. P. Feinberg, K. D. Hansen and R. A. Irizarry (2014). "Minfi: a flexible and comprehensive Bioconductor package for the analysis of Infinium DNA methylation microarrays." Bioinformatics **30**(10): 1363-1369.

[2] Barillot, E., F. Radvanyi, J.-P. Thiery, N. Stransky and P. Hupé (2004). "Analysis of array CGH data: from signal ratio to gain and loss of DNA regions." Bioinformatics **20**(18): 3413-3422.

[3] Cirenajwis, H., H. Ekedahl, M. Lauss, K. Harbst, A. Carneiro, J. Enoksson, F. Rosengren, L. Werner-Hartman, T. Törngren, A. Kvist, et al. (2015). "Molecular stratification of metastatic melanoma using gene expression profiling: Prediction of survival outcome and benefit from molecular targeted therapy." Oncotarget **6**(14): 12297-12309.

[4] Fortin, J.-P., T. J. Triche, Jr. and K. D. Hansen (2017). "Preprocessing, normalization and integration of the Illumina HumanMethylationEPIC array with minfi." Bioinformatics (Oxford, England) **33**(4): 558-560.

[5] Harbst, K., M. Lauss, H. Cirenajwis, C. Winter, J. Howlin, T. Törngren, A. Kvist, B. Nodin, E. Olsson, J. Häkkinen, et al. (2014). "Molecular and genetic diversity in the metastatic process of melanoma." The Journal of pathology **233**(1): 39-50.

[6] Lauss, M., R. Haq, H. Cirenajwis, B. Phung, K. Harbst, J. Staaf, F. Rosengren, K. Holm, M. Aine, K. Jirström, et al. (2015). "Genome-Wide DNA Methylation Analysis in Melanoma Reveals the Importance of CpG Methylation in MITF Regulation." Journal of Investigative Dermatology **135**(7): 1820-1828.

[7] Li, J., R. Lupat, K. C. Amarasinghe, E. R. Thompson, M. A. Doyle, G. L. Ryland, R. W. Tothill, S. K. Halgamuge, I. G. Campbell and K. L. Gorringe (2012). "CONTRA: copy number analysis for targeted resequencing." Bioinformatics **28**(10): 1307-1313.

[8] McCartney, D. L., R. M. Walker, S. W. Morris, A. M. McIntosh, D. J. Porteous and K. L. Evans (2016). "Identification of polymorphic and off-target probe binding sites on the Illumina Infinium MethylationEPIC BeadChip." Genomics data **9**: 22-24.

[9] Monti, S., P. Tamayo, J. Mesirov and T. Golub (2003). "Consensus Clustering: A Resampling-Based Method for Class Discovery and Visualization of Gene Expression Microarray Data." Machine Learning **52**(1): 91-118.

[10] Morris, T. J., L. M. Butcher, A. Feber, A. E. Teschendorff, A. R. Chakravarthy, T. K. Wojdacz and S. Beck (2014). "ChAMP: 450k Chip Analysis Methylation Pipeline." Bioinformatics **30**(3): 428-430.

[11] Orozco, J. I. J., T. A. Knijnenburg, A. O. Manughian-Peter, M. P. Salomon, G. Barkhoudarian, J. R. Jalas, J. S. Wilmott, P. Hothi, X. Wang, Y. Takasumi, et al. (2018). "Epigenetic profiling for the molecular classification of metastatic brain tumors." Nature Communications **9**(1): 4627.

[12] R Core Team. (2016). "R: A Language and Environment for Statistical Computing." from <https://www.R-project.org/>.

[13] Reinius, L. E., N. Acevedo, M. Joerink, G. Pershagen, S.-E. Dahlén, D. Greco, C. Söderhäll, A. Scheynius and J. Kere (2012). "Differential DNA Methylation in Purified Human Blood Cells: Implications for Cell Lineage and Studies on Disease Susceptibility." PLOS ONE **7**(7): e41361.

[14] Teschendorff, A. E., F. Marabita, M. Lechner, T. Bartlett, J. Tegner, D. Gomez-Cabrero and S. Beck (2013). "A beta-mixture quantile normalization method for correcting probe design bias in Illumina Infinium 450 k DNA methylation data." Bioinformatics (Oxford, England) **29**(2): 189-196.

[15] Triche, T. J., Jr., D. J. Weisenberger, D. Van Den Berg, P. W. Laird and K. D. Siegmund (2013). "Low-level processing of Illumina Infinium DNA Methylation BeadArrays." Nucleic acids research **41**(7): e90-e90.

**Supplementary Figures:**

**Fig S1:** *Additional characteristics of Lund immune-methylation clusters.* A) Consensus clustering plot for immune-methylation clusters. B) Forest plot of multivariate Cox regression analysis adjusted for gender and age groups using DMFS for clusters (Only regional lymph node metastases). C) Forest plot with adjustments for metastasis type and using DSS for clusters (All metastases except local recurrences). D) Barplot of correlation between methylation and corresponding gene expression for the selected immune CpG set. E) Kaplan-Meier plot for association between different TILs IHC categories (**Absent** (*Absence of infiltration*), **Non-brisk** (*localized infiltration*) and **Brisk** (*Strong infiltration*)) and DSS for Lund cohort. Numbers at risk and percentages at risk (within brackets) are indicated below each plot. P-values calculated using log-rank test. Maximum time to display on x-axis has been clipped to 10 years.

**Fig S2:** *Additional characteristics of immune exclusion in the immune-methylation clusters.* A) Boxplot showing distribution of c-MYC (*MYC*) mRNA expression across Lund clusters. P-value calculated using Kruskal-Walis test. B) Boxplot showing distribution of Beta-Catenin (*CTNNB1*) mRNA expression for MITF^-^ samples across clusters (Left) and same for MITF^+^ samples (right). P-values from Kruskal-Walis test. C) Boxplot showing distribution of *PIK3R1* mRNA expression across Lund clusters. P-value calculated using Kruskal-Walis test. D) Venn diagram showing intersection among molecular events of *PTEN* gene in the same samples. E) Boxplot showing distribution of *PTEN* mRNA expression for samples with and without any *PTEN* alteration event. P-value from Wilcoxon rank-sum (Mann-Whitney) test. A PTEN alteration event is defined as promoter hypermethylation, non-synonymous mutation or copy number loss/deletion. F) Boxplot showing distribution of *PTEN* mRNA expression across TILs IHC categories (**Absent** (*Absence of infiltration*), **Non-brisk** (*localized infiltration*) and **Brisk** (*Strong infiltration*)) for Lund tumors. G) Barplot showing distribution of overall *PTEN* alteration event status across TCGA clusters.

**Fig S3:** *Additional characteristics of TCGA immune-methylation clusters.* A) Heatmap of methylation profile along centroid CpGs for TCGA immune-methylation clusters. Color bar on the bottom-right indicates the corresponding range for methylation β. Color bars on the top-left indicate the corresponding cell type for the CpG. Row names indicate corresponding gene names for the CpGs. B) Oncoplot of important melanoma associated mutations across TCGA immune-methylation clusters. Color bar on the bottom-right indicates the mutation category. Hotspot mutations are defined to involve V600, K601 for BRAF and Q61, G12, G13 for RAS. C) Forest plot with adjustments for metastasis type and using OS for TCGA clusters (all metastases except local recurrences).

Table S1: List of immune cell type associated CpGs with corresponding gene and associated cell type information

| **CpG id** | **Symbol** | **Chromosome** | **CpG feature^a^** | **Cell type** | **Centroid CpG^b^** |
| --- | --- | --- | --- | --- | --- |
| cg15258980 | ARHGAP25 | 2 | TSS1500 | Activated B cell | Yes |
| cg04332373 | CD38 | 4 | TSS1500 | Activated B cell | Yes |
| cg15742700 | BLK | 8 | TSS1500 | Activated B cell | No |
| cg05433111 | CD19 | 16 | TSS200 | Activated B cell | Yes |
| cg09648933 | CD79B | 17 | TSS200 | Activated B cell | Yes |
| cg17934790 | CLEC17A | 19 | TSS200 | Activated B cell | Yes |
| cg07979271 | SPIB | 19 | TSS1500 | Activated B cell | Yes |
| cg07571745 | LCK | 1 | TSS1500 | Activated CD8 T cell | Yes |
| cg10628126 | GNLY | 2 | TSS200 | Activated CD8 T cell | Yes |
| cg19410791 | CD8A | 2 | TSS1500 | Activated CD8 T cell | Yes |
| cg12916723 | NKG7 | 19 | TSS1500 | Activated CD8 T cell | No |
| cg11362935 | POU2AF1 | 11 | TSS200 | B cell | Yes |
| cg19599951 | CXCR5 | 11 | TSS1500 | B cell | Yes |
| cg07667560 | POU2F2 | 19 | TSS1500 | B cell | No |
| cg16454902 | IL21R | 16 | TSS200 | CD56 dim natural killer cell | Yes |
| cg08422803 | ITGB2 | 21 | TSS200 | Central memory CD4 T cell | Yes |
| cg02543462 | IL1RN | 2 | TSS200 | Central memory CD8 T cell | Yes |
| cg10798745 | CD160 | 1 | TSS200 | Effector memory CD8 T cell | Yes |
| cg07623567 | HLA-DMB | 6 | TSS1500 | Effector memory CD8 T cell | Yes |
| cg21201401 | LIME1 | 20 | TSS200 | Effector memory CD8 T cell | Yes |
| cg17658002 | ACP5 | 19 | TSS1500 | Gamma delta T cell | Yes |
| cg04331601 | FAM129C | 19 | TSS1500 | Immature B cell | Yes |
| cg15743985 | CD22 | 19 | TSS1500 | Immature B cell | Yes |
| cg19595244 | AHCYL1 | 1 | TSS1500 | Immature dendritic cell | Yes |
| cg15246085 | AMT | 3 | TSS200 | Immature dendritic cell | No |
| cg13448978 | FGR | 1 | TSS200 | Macrophage | Yes |
| cg18468844 | PTAFR | 1 | TSS1500 | Macrophage | Yes |
| cg14851700 | GLUL | 1 | TSS1500 | Macrophage | No |
| cg13108255 | NCF2 | 1 | TSS1500 | Macrophage | Yes |
| cg24170465 | FAM198B | 4 | TSS200 | Macrophage | No |
| cg24211388 | AIF1 | 6 | TSS1500 | Macrophage | Yes |
| cg24964103 | VNN1 | 6 | TSS200 | Macrophage | Yes |
| cg18589858 | SLCO2B1 | 11 | TSS1500 | Macrophage | No |
| cg09774932 | WNT5B | 12 | TSS1500 | Macrophage | Yes |
| cg22485810 | ADAP2 | 17 | TSS200 | Macrophage | Yes |
| cg21495704 | TYROBP | 19 | TSS200 | Macrophage | No |
| cg10451565 | GPR77 | 19 | TSS1500 | Macrophage | No |
| cg13424229 | CPA3 | 3 | TSS1500 | Mast cell | No |
| cg06350097 | FCGR2A | 1 | TSS1500 | MDSC | No |
| cg14654385 | FERMT3 | 11 | TSS1500 | MDSC | Yes |
| cg09383860 | IL18BP | 11 | TSS200 | MDSC | Yes |
| cg15337006 | ITGAM | 16 | TSS1500 | MDSC | Yes |
| cg12555844 | PARVG | 22 | TSS1500 | MDSC | Yes |
| cg06983746 | FASLG | 1 | TSS200 | Natural killer cell | Yes |
| cg18563860 | CTSZ | 20 | TSS1500 | Natural killer cell | Yes |
| cg08058560 | CD101 | 1 | TSS200 | Natural killer T cell | Yes |
| cg12852800 | CNPY3 | 6 | TSS1500 | Natural killer T cell | No |
| cg03154580 | RALB | 2 | TSS1500 | Plasmacytoid dendritic cell | Yes |
| cg20810975 | ITGA2B | 17 | TSS1500 | Plasmacytoid dendritic cell | Yes |
| cg02792780 | STAB1 | 3 | TSS200 | Regulatory T cell | Yes |
| cg13409077 | CD72 | 9 | TSS200 | Regulatory T cell | Yes |
| cg01004382 | STAT4 | 2 | TSS200 | T cell | Yes |
| cg07930752 | CD28 | 2 | TSS1500 | T cell | Yes |
| cg02374486 | PRF1 | 10 | TSS1500 | T cell | Yes |
| cg06970090 | SPOCK2 | 10 | TSS200 | T cell | Yes |
| cg18637238 | KLRK1 | 12 | TSS1500 | T cell | No |
| cg15676719 | RCSD1 | 1 | TSS1500 | Type 1 T helper cell | Yes |
| cg00461022 | FAM134B | 5 | TSS1500 | Type 1 T helper cell | Yes |
| cg14674856 | LTC4S | 5 | TSS1500 | Type 1 T helper cell | No |
| cg05992079 | SIT1 | 9 | TSS200 | Type 1 T helper cell | Yes |
| cg04517263 | TRAF1 | 9 | TSS200 | Type 1 T helper cell | Yes |
| cg00805360 | ADAM8 | 10 | TSS1500 | Type 1 T helper cell | No |
| cg20186396 | CD7 | 17 | TSS200 | Type 1 T helper cell | Yes |
| cg18738581 | IL17RE | 3 | TSS200 | Type 17 T helper cell | Yes |
| cg24065504 | ANKRD22 | 10 | TSS1500 | Type 17 T helper cell | No |
| cg04982834 | EVI5 | 1 | TSS1500 | Type 2 T helper cell | Yes |
| cg08450017 | CXCR6 | 3 | TSS200 | Type 2 T helper cell | Yes |

^a^ TSS200 and TSS1500: Within 200 and 1500 base pair distance from the transcription start site respectively

^b^ Centroid CpG column indicates whether the CpG was used for the construction of methylation centroids

Table S2: Clinical characteristics for immune-methylation clusters (Lund cohort)

|  | Entire cohort | Cluster 1 | Cluster 2 | Cluster 3 | P value |
| --- | --- | --- | --- | --- | --- |
| Patient characteristics | | | | | |
| Gender n (%) |  | | | | |
| Male | 108 (60) | 45 (62) | 23 (56) | 40 (61) | 0.83 |
| Female | 72 (40) | 28 (38) | 18 (44) | 26 (39) |  |
|  | | | | | |
| Age at diagnosis median (range) | 63.5 (22-91) | 64 (29-88) | 65 (33-89) | 62.5 (22-91) | 0.28 |
|  | | | | | |
| Tumor characteristics | | | | | |
| Location n (%) |  | | | | |
| Cutaneous | 6 (3) | 1 (1) | 0 (0) | 5 (8) | 0.03 |
| Lymph node | 123 (68) | 59 (80) | 28 (68) | 36 (55) |  |
| Other ^a^ | 1 (0) | 1 (1) | 0 (0) | 0 (0) |  |
| Subcutaneous | 29 (16) | 8 (11) | 5 (12) | 16 (24) |  |
| Visceral | 8 (4) | 2 (3) | 3 (7) | 3 (5) |  |
| *NA* | 13 (7) | 2 (3) | 5 (12) | 6 (9) |  |
|  | | | | | |
| Metastasis type n (%) |  | | | | |
| Local recurrence | 8 (4) | 3 (4) | 0 (0) | 5 (8) | 0.007 |
| Regional lymph node | 94 (52) | 49 (67) | 21 (51) | 24 (36) |  |
| Regional other ^b^ | 17 (9) | 4 (5) | 3 (7) | 10 (15) |  |
| Distant Metastasis | 54 (30) | 15 (21) | 14 (34) | 25 (38) |  |
| *NA* | 7 (4) | 2 (3) | 3 (7) | 2 (3) |  |
|  | | | | | |
| Primary tumor characteristics | | | | | |
| ALM | 5 (3) | 1 (1) | 0 (0) | 4 (6) | - |
| Mucosal | 1 (0) | 0 (0) | 1(2) | 0 (0) |  |
| NM | 64 (36) | 27 (37) | 15 (37) | 22 (33) |  |
| Other ^c^ | 3 (2) | 2 (3) | 1(2) | 0 (0) |  |
| SSM | 39 (22) | 16 (22) | 11 (27) | 12 (18) |  |
| Unknown primary | 24 (13) | 8 (11) | 6 (15) | 10 (15) |  |
| *NA* | 44 (24) | 19 (26) | 7 (17) | 18 (27) |  |

^a c^ Melamonas with unknown origin

^b^ Includes cutaneous, subcutaneous and in-transit metastases

Table S3: Overlap between Lund gene expression phenotypes and Lund immune-methylation clusters.

|  | Lund immune-methylation clusters | | | |
| --- | --- | --- | --- | --- |
| **Lund**  **gene**  **expression**  **phenotypes** |  | **Cluster 1**  (Low immune-methylation)  **n (%)** | **Cluster 2**  (Intermediate immune-methylation)  **n (%)** | **Cluster 3**  (High immune-methylation)  **n (%)** |
|  | **High-Immune** | 17 (43) | 7 (22) | 2 (4) |
|  | **Normal-like** | 1 (3) | 0 (0) | 1 (2) |
|  | **Pigmentation** | 15 (38) | 16 (50) | 35 (61) |
|  | **Proliferative** | 3 (8) | 6 (19) | 17 (30) |
|  | **Unclassified**^a^ | 4 (10) | 3 (9) | 2 (4) |

^a^ Samples which cannot be classified into one of the four gene expression phenotypes

Table S4: List of centroid CpGs with methylation β values for each cluster

| **CpG id** | **Cluster 1** | **Cluster 2** | **Cluster 3** |
| --- | --- | --- | --- |
| cg15258980 | 0.5 | 0.78 | 0.94 |
| cg04332373 | 0.42 | 0.62 | 0.77 |
| cg05433111 | 0.71 | 0.82 | 0.85 |
| cg09648933 | 0.51 | 0.66 | 0.78 |
| cg17934790 | 0.73 | 0.79 | 0.87 |
| cg07979271 | 0.86 | 0.93 | 0.94 |
| cg07571745 | 0.4 | 0.64 | 0.79 |
| cg10628126 | 0.85 | 0.85 | 0.91 |
| cg19410791 | 0.72 | 0.86 | 0.96 |
| cg11362935 | 0.49 | 0.59 | 0.72 |
| cg19599951 | 0.77 | 0.95 | 0.96 |
| cg16454902 | 0.25 | 0.39 | 0.43 |
| cg08422803 | 0.51 | 0.76 | 0.9 |
| cg02543462 | 0.51 | 0.62 | 0.79 |
| cg10798745 | 0.59 | 0.76 | 0.87 |
| cg07623567 | 0.3 | 0.58 | 0.66 |
| cg21201401 | 0.35 | 0.72 | 0.94 |
| cg17658002 | 0.61 | 0.73 | 0.86 |
| cg04331601 | 0.6 | 0.71 | 0.84 |
| cg15743985 | 0.65 | 0.8 | 0.92 |
| cg19595244 | 0.89 | 0.77 | 0.89 |
| cg13448978 | 0.73 | 0.78 | 0.9 |
| cg18468844 | 0.94 | 0.96 | 0.98 |
| cg13108255 | 0.6 | 0.76 | 0.83 |
| cg24211388 | 0.86 | 0.83 | 0.92 |
| cg24964103 | 0.91 | 0.91 | 0.95 |
| cg09774932 | 0.33 | 0.47 | 0.64 |
| cg22485810 | 0.4 | 0.53 | 0.69 |
| cg14654385 | 0.77 | 0.78 | 0.87 |
| cg09383860 | 0.56 | 0.73 | 0.86 |
| cg15337006 | 0.76 | 0.78 | 0.9 |
| cg12555844 | 0.58 | 0.65 | 0.78 |
| cg06983746 | 0.7 | 0.85 | 0.91 |
| cg18563860 | 0.69 | 0.82 | 0.91 |
| cg08058560 | 0.54 | 0.78 | 0.96 |
| cg03154580 | 0.77 | 0.79 | 0.86 |
| cg20810975 | 0.65 | 0.76 | 0.87 |
| cg02792780 | 0.51 | 0.54 | 0.68 |
| cg13409077 | 0.51 | 0.59 | 0.69 |
| cg01004382 | 0.51 | 0.77 | 0.93 |
| cg07930752 | 0.73 | 0.86 | 0.89 |
| cg02374486 | 0.55 | 0.71 | 0.83 |
| cg06970090 | 0.42 | 0.51 | 0.62 |
| cg15676719 | 0.49 | 0.64 | 0.78 |
| cg00461022 | 0.71 | 0.89 | 0.97 |
| cg05992079 | 0.39 | 0.67 | 0.8 |
| cg04517263 | 0.46 | 0.72 | 0.93 |
| cg20186396 | 0.59 | 0.67 | 0.76 |
| cg18738581 | 0.59 | 0.73 | 0.85 |
| cg04982834 | 0.83 | 0.84 | 0.91 |
| cg08450017 | 0.65 | 0.85 | 0.94 |

Table S5: Overlap between TCGA gene expression classes and TCGA immune-methylation clusters.

|  | TCGA immune-methylation clusters | | | |
| --- | --- | --- | --- | --- |
| **TCGA**  **gene**  **expression**  **classes** |  | **Cluster 1**  (Low immune-methylation)  **n (%)** | **Cluster 2**  (Intermediate immune-methylation)  **n (%)** | **Cluster 3**  (High immune-methylation)  **n (%)** |
|  | **Immune** | 74 (93) | 44 (65) | 42 (25) |
|  | **Keratin** | 6 (7) | 17 (25) | 77 (45) |
|  | **MITF-low** | 0 (0) | 5 (7) | 52 (30) |
|  | ***NA***^a^ | 0(0) | 2 (3) | 0 (0) |

^a^ Samples for which TCGA gene expression class information was unavailable
